# Copy number variations in endoglin locus: mapping of large deletions in Spanish families with hereditary hemorrhagic telangiectasia type 1

**DOI:** 10.1186/1471-2350-14-121

**Published:** 2013-11-25

**Authors:** Ana Fontalba, Jose L Fernández-Luna, Roberto Zarrabeitia, Lucia Recio-Poveda, Virginia Albiñana, Maria L Ojeda-Fernández, Carmelo Bernabéu, Luis A Alcaraz, Luisa M Botella

**Affiliations:** 1 Molecular Genetics Unit, Hospital Valdecilla, and Instituto de Formación e Investigación Marqués de Valdecilla (IFIMAV), Santander, Spain; 2 HHT Spanish Unit, Hospital Sierrallana and Centro de Investigacion Biomedica en Red de Enfermedades Raras (CIBERER), Torrelavega, Santander, Spain; 3 Centro de Investigaciones Biológicas, Consejo Superior de Investigaciones Científicas (CSIC), and Centro de Investigación Biomédica en Red de Enfermedades Raras (CIBERER), Madrid, Spain; 4 Bioarray S.L, Crevillente, Alicante, Spain

**Keywords:** Hereditary hemorrhagic telangiectasia (HHT), Endoglin deletions, Multiplex ligation PCR assisted assay (MLPA), Copy number variation (CNV) arrays, Alu repetitive sequences

## Abstract

**Background:**

The hereditary hemorrhagic telangiectasia syndrome (HHT), also known as the Rendu–Osler-Weber syndrome is a multiorganic vascular disorder inherited as an autosomal dominant trait. Diagnostic clinical criteria include: epistaxis, telangiectases in mucocutaneous and gastrointestinal sites, arteriovenous malformations (AVMs) most commonly found in pulmonary, hepatic and cerebral circulations, and familial inheritance. HHT is transmitted in 90% of the cases as an autosomal dominant condition due to mutations in either endoglin (*ENG*), or activin receptor-like kinase 1 (ACVRL1/ALK1) genes (HHT type 1 and 2, respectively).

**Methods:**

We have carried out a genetic analysis of four independent Spanish families with HHT clinical criteria, which has permitted the identification of new large deletions in ENG. These mutations were first detected using the MLPA technique and subsequently, the deletion breakpoints were mapped using a customized copy number variation (CNV) microarray. The array was designed to cover the ENG gene and surrounding areas.

**Results:**

All tested families carried large deletions ranging from 3-kb to 100-kb, involving the *ENG* gene promoter, several ENG exons, and the two downstream genes FGSH and CDK9. Interestingly, common breakpoints coincident with Alu repetitive sequences were found among these families.

**Conclusions:**

The systematic hybridization of DNA from HHT families, with deletions or duplications, to custom designed microarrays, could allow the mapping of breakpoints, coincident with repetitive Alu sequences that might act as “hot spots” in the development of chromosomal anomalies.

## Background

The hereditary hemorrhagic telangiectasia syndrome (HHT), also known as Rendu–Osler-Weber syndrome [1-3] is a vascular disorder inherited as an autosomal dominant trait. Careful epidemiological studies have revealed that HHT affects approximately 1 in 5,000 individuals [4,5] and is therefore considered to be an inherited rare vascular disease.

The clinical symptoms characteristic of HHT are the so-called Curaçao criteria [2], which help in its diagnosis when at least 3 of the 4 criteria are present in a patient. These include: epistaxis (nose bleeds), telangiectasia at mucocutaneous and gastrointestinal sites, arteriovenous malformations (AVMs) most commonly found in pulmonary, hepatic and cerebral circulations, and dominant familial inheritance [3,6].

HHT is transmitted as an autosomal dominant condition due to a single mutation in *Endoglin* (*ENG*; HHT1) [7], *Activin Receptor-Like Kinase 1 *(*ACVRL1/ALK1*; HHT2) [8], or *MADH4/SMAD4* (JHPT, a combined syndrome of juvenile polyposis and HHT) [9]. The involvement of all these genes in the transforming growth factor (TGF-β) signaling pathway is inherent in HHT pathogenesis [10]. There are at least two further unidentified genes that can cause HHT: HHT3 between 141.9 and 146.4 Mb on chromosome 5q [3,11] and HHT4 on chromosome 7p between D7S2252 and D7S510.130 [12].

The genes mutated in HHT encode proteins that mediate signaling by the TGF-β superfamily. Members of this superfamily such as TGF-βs, bone morphogenetic proteins (BMPs), activins, nodals, growth/differentiation factors (GDFs) and inhibins regulate diverse cellular functions by binding to a heteromeric complex of type I and type II transmembrane serine/threonine kinase receptors [13]. In the TGF-β signaling cascade, the type II receptor with very high ligand affinity, co-operatively recruits and transphosphorylates the type I receptor by direct contact with the ligand-modified N-terminus of TβRI [14]. In Smad-dependent TGF-β pathways, the type I receptor subsequently phosphorylates and activates receptor-associated (R)-Smads, according to the receptor complex involved. R-Smads bind to Smad4 and translocate to the nucleus where they influence transcriptional activity with co-activators and co-repressors.

In endothelial cells, upon ligand binding, TβRII can associate with two different TGF-β type I receptors ALK-5 or ALK-1 [15]. Endoglin is an auxiliary receptor that modulates both associations in an opposite manner. Thus, while endoglin promotes signaling through ALK-1, it inhibits the ALK-5 pathway [16,17]. In turn, ALK-1 and ALK-5 activate distinct Smad pathways, resulting in opposing endothelial cell responses in terms of proliferation, migration, and pro- or anti-angiogenic gene expression [15,18,19].

So far, more than 600 different mutations have been found in *ENG* and *ACVRL1* in HHT families (HHT mutation database; http://www.arup.utah.edu/database/hht/). Mutations range from single base-pair changes to major deletions of multiple exons. Recently, pathogenic mutations affecting the 5’ UTR region of *ENG* leading to new translation initiation sites (TIS) dominant over the normal endoglin TIS have also been described [20,21].

In the present study we describe for the first time, a series of 4 families, harboring 4 different independent mutations in the *ENG* gene, not described so far in literature. All of them were large deletions starting in the 5’ upstream region of *ENG,* and spanning just the promoter region, one or several exons, or even a big 100-Kb deletion encompassing the whole *ENG* gene and the two downstream genes *FGSH* and *CDK9*. These mutations were first detected by MLPA technique, and subsequently the breakpoints were characterized using a customized copy number variation (CNV) microarray, designed to cover *ENG* and flanking sequences. Interestingly, common breakpoints located within “Alu” sequences were found among these families. To the best of our knowledge, this is the first time a fine mapping of a series of deletion mutations is described in HHT taking advantage of a customized CNV/CGH microarray.

## Methods

### Patient samples

Blood samples of 4 independent families from Spain with clinically confirmed HHT (the presence of three or four Curaçao criteria) were subjected to molecular diagnostics. The clinical data of these patients were obtained from screening performed in the Spanish HHT reference Center of Sierrallana, or alternatively from the referring physicians. Informed consent was obtained from all the patients.

### Ethics statement request

Human blood samples and clinical data reported in this manuscript were obtained with the prior approval of the appropriate ethics committees of the CSIC for the research conducted in the Centro de Investigaciones Biológicas (CIB, Madrid), the CEIC (Clinical Research Ethics Committee) of the Cantabrian Health Service for patients attending the Hospital of Sierrallana (Torrelavega, Santander, Cantabria), and the Medical Genetics department of the Hospital Valdecilla (Santander, Cantabria). Research was carried out in compliance with the Helsinki Declaration ( http://www.wma.net/en/30publications/10policies/b3/index.html), keeping the results strictly confidential, with number codes for the identification of patients.

### DNA extraction and mutation analysis

DNA was extracted from peripheral blood using standard procedures. Each coding exon and its flanking intronic sequences were amplified by PCR and sequenced for each of the two genes (*ENG* and *ACVRL1*). Primer sequences and PCR conditions have already been reported [10,22] and are available upon request. PCR products were run on 1.5% agarose/1 × TAE gels and purified using a PCR purification kit (Millipore, Germany). The PCR products were then sequenced in forward and reverse orientation on an Applied Biosystems sequencer using the dye terminator cycle sequencing kit according to the manufacturer’s instructions. If no mutation was found either in *ENG* or *ALK1*, MLPA was performed. DNA concentration was measured for each sample using a Nanodrop spectrophotometer. At the same time the DNA integrity was checked by agarose gel electrophoresis (0.8% in TAE).

### Multiplex ligation-dependent probe amplification (MLPA)

MLPA [23] was performed with 200 ng of genomic DNA according to the manufacturer’s instructions using the P093 Salsa MLPA HHT/PPH1 probe set (MRC-Holland, Amsterdam, The Netherlands). Probe amplification products were run on an ABI PRISM 310 Genetic Analyzer using GeneScan-500 TAMRA Size Standard (PE Applied Biosystems). Data were analyzed using Coffalyser MLPA data analysis software, and a relative copy number was obtained after normalization of peaks against controls. Values between 0.75 and 1.3 were considered to be within the normal range. (LLC, State College, PA). Because of variations in assay performance, we used dosage ratio values of ≤0.7 as our boundaries.

### Characterization of the breakpoints

**Figure 1:**
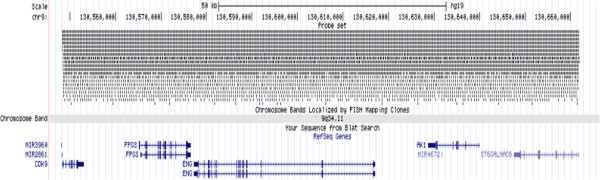
**Schematic representation of the probes covering the *ENG *gene and its flanking regions on the human chromosome 9.** The probes overlap centered at *ENG* (tiling microarray).

Breakpoints were studied using a custom designed CGH microarray (Agilent) to detect copy number variations. A tiling microarray was designed with eArray (Agilent). The array contains a total of 15,744 probes, including: i) 7,568 probes of *ENG*; ii) 3,927 probes from the rest of chromosome 9; iii) 1,262 probes included for normalization; and iv) 2,987 probes as Agilent controls. A total of 7,568 probes were selected in the region of interest (chr9: 130548305–130661871, hg19) covering the *ENG* gene and the surrounding upstream and downstream sequences to complete a total area of 113-Kb, with the *ENG* gene in its center (Figure 1). Probes were designed with an average probe spacing of 15-bp, and allowed to vary in size (from 45- to 60-mer) in order to maintain their Tm about 80°C. The rest of the chromosome 9 was covered with 3,927 additional probes. For normalization purposes during data analysis, 1,262 probes, distributed among all chromosomes, were also included. 2,987 probes were Agilent controls. A probe performance score was assigned to each probe by the software.

Labeling was carried out according to manufacturer protocol l (Agilent Oligonucleotide Array-Based CGH for Genomic DNA Analysis, v6.3). Briefly, eight genomic DNA samples, including four controls (non HTT patients), were Cy5 labeled and co-hybridized with a sex matched, Cy3 labeled human genomic reference DNA (Promega). After hybridization, arrays were washed and the signal for each probe was captured (scanned) with an Agilent scanner. Images were analyzed using Agilent Feature Extraction v10.7 software. The quality of the hybridization was estimated by the DLRS parameter, indicating the dispersion among the hybridization signaling of the probes. In all cases, good DLRS values, between 0.1 and 0.2 were obtained.

Finally, raw data were analyzed with Agilent Genomic Workbench 6.5. Pre-processing steps were done by applying GC correction and centralization. Probes with scores below 0.5 were filtered out. Aberration calling was done with the ADM-2 algorithm with a threshold of 0.6. An aberration was considered when there were at least 5 consecutives probes with a minimum absolute average log ratio of 0.25. The breakpoint regions were inspected with a UCSC Genome Browser, using the Repeat Masker database to identify repeated elements.

## Results

### Correlation between the presence of deletions in *ENG* and the clinical phenotype

Table 1 shows the clinical symptoms and genetics of HHT patients belonging to 4 independent families. The clinical symptoms are always accompanied by the presence of the mutations commented on below. Those patients with known genetics, but referenced in Table 1 with “no data” for the different clinical HHT symptoms, had not undergone a complete clinical screening, whereas patients with complete data were either, screened in the Spanish HHT Reference Center of Sierrallana, or elsewhere. In these families, there was a perfect phenotype-genotype correlation between clinical criteria and the finding of mutations in *ENG.* Exceptions to this rule were children and teen-agers, most of them without relevant epistaxis, with no telangiectases, and not subjected to the complete HHT protocol of clinical screening. Close relatives with no clinical symptoms were also genetically tested.

**Table 1:** Genotype-phenotype correlation in 4 families with HHT

**Family**	**Epistaxis**	**Telangiect**	**PAVMs**	**CAVMs**	**HAVMs**	**GB**	**Genetics**
**NMEx**							
JCG	**Yes**	**Yes**	**Yes**	No	No	No	Δ Promoter ENG
PCG	**Yes**	**Yes**	No	No	No	No	Δ Promoter ENG
MJCS	**Yes**	**Yes**	**Yes**	No	No	No	Δ Promoter ENG
VAC	**Yes**	**Yes**	No	No	No	No	Δ Promoter ENG
**198**							
198/01/11	**Yes**	**Yes**	**Yes**	No	No	No	Δ Promoter + exon 1 ENG
198/02/11	**Yes**	**Yes**	No data	No data	No data	No data	Δ Promoter + exon 1 ENG
198/03/11 3 y	No	No	No data	No data	No data	No data	Δ Promoter + exon 1 ENG
**71**							
71/02/08	**Yes**	**Yes**	**Yes**	No	**Yes**	**Yes**	Δ Promoter + exons 1-2-3 ENG
71/03/11	No data	No data	No data	No data	No data	**Yes**	Δ Promoter + exons 1-2-3 ENG
71/04/11	No data	No data	No data	No data	No data	No data	Δ Promoter + exons 1-2-3 ENG
71/08/1113 y	No	No data	**Yes**	No data	No data	No data	Δ Promoter + exons 1-2-3 ENG
71/09/1114 y	**Yes**	No data	**Yes**	No data	No data	No data	Δ Promoter + exons 1-2-3 ENG
71/12/11 6 y	No	No data	No data	No data	No data	No data	Δ Promoter + exons 1-2-3 ENG
**GUM**							
MCDV	**Yes**	**Yes**	**Yes**	Yes*	No	No	#Promoter and complete allele ENG (−9 kb + all exons)
JMVD	**Yes**	**Yes**	No data	No data	No data	No data	#Promoter and complete allele ENG (−9 kb + all exons)
JDHV	**Yes**	**Yes**	No data	No data	No data	No data	#Promoter and complete allele ENG (−9 kb + all exons)

E, stands for epistaxis, and T for telangiectases. PAVM, CAVM, HAVM stand for pulmonary, cerebral and hepatic arteriovenous malformations, respectively.

GB means gastric bleeding. *patient MCDV had a brain abscess in addition to CAVMs. Δ stands for deletion. # stands for complete deletion of gene.

### Family NMEx

**Figure 2:**
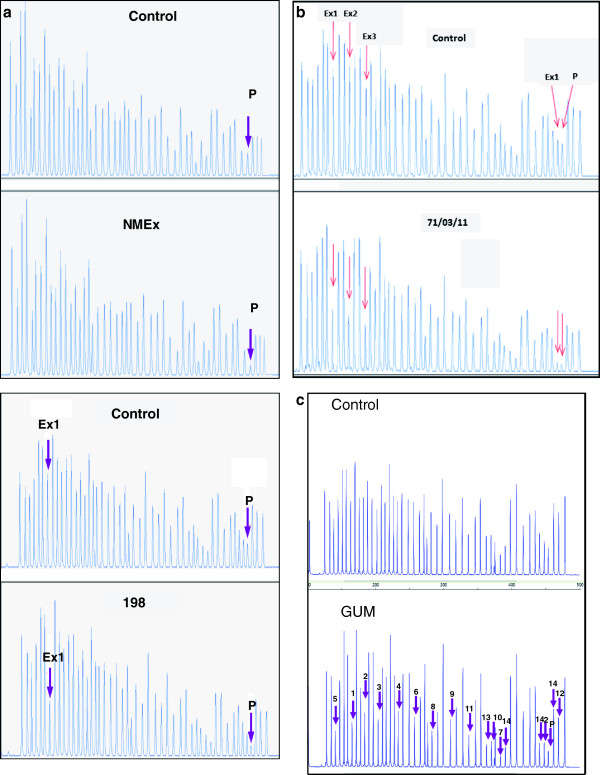
**MLPA results of the 4 different families analyzed in the present work. A)** Control and chromatogram representing the family with promoter deletion (NMEx) and with promoter and exon 1 deletion (198). **B)** Control chromatogram and family 71 exhibiting deletion of promoter and exons from 1–3. **C)** Family GUM with whole deletion of *ENG* gene including the promoter. In the chromatograms the height of the peaks is proportional to the amplification of the corresponding exon probe. The affected exons are indicated by arrows in each case.

Family NMEx, comes from Navalmoral de la Mata, a town in Extremadura, western Spain. These patients were referred to our HHT unit from the local hospital of this town, because they presented epistaxis, telangiectasia, and some of them also suffered from internal arteriovenous malformations (Table 1). After amplification and sequencing of all exons and intron-exon boundaries of *ENG* and *ACVRL1* genes, no mutation was found. Therefore, according to the protocol established in our HHT unit, an MLPA was performed. By comparing patient JCG with a control sample (Figure 2A), it can be seen that only the peak corresponding to the *ENG* MLPA probe annealing in the proximal promoter region appears affected. The same result was obtained for the four members of this family, whose clinical data are listed in Table 1, while relatives without HHT criteria, did not show any alteration in the MLPA (data not shown). Notably, no mutation involving a deletion of the *ENG* promoter has been reported in HHT so far. The possible existence of a polymorphism in the hybridization area of the corresponding MLPA probe, which could prevent primer annealing, was discarded by sequencing the proximal promoter region, from -450 bp to the transcription start (data not shown).

### Family 198

In this family from northern Spain, a total of 3 members with clinical diagnosis of HHT were subjected to the normal protocol of PCR amplification and sequencing, but no mutations in either *ENG* or *ACVRL1* were observed. As in the case of family NMEx, MLPA analysis was carried out, and a deletion involving the promoter and exon 1 of *ENG* was found. In Figure 2A, bottom panel, a representative chromatogram of an affected family member (198/01/11) reveals that peaks corresponding to promoter and exon 1 are both decreased in comparison with a control DNA.

### Family 71

A total of 6 members, 3 adults and 3 children, were genetically screened. All three adults fulfilled the clinical Curaçao criteria. As in previous families, while the PCR and sequencing did not reveal any point mutation or short duplication/deletion, the MLPA showed a large deletion encompassing the promoter and exons 1 to 3 of ENG (Figure 2B). Moreover, the 3 children had no visible clinical signs of HHT, but their positive genetic test result prompted a subsequent, in depth clinical examination and led to the finding of pulmonary AVMs in two of them.

### Family GUM

Three members of a family from Madrid were diagnosed with clinical criteria of HHT. The conventional genetic analysis did not find any mutation, but MLPA analysis (Figure 2C), revealed the complete absence of one *ENG* allele starting at 9 kb upstream from the transcription start site, suggesting that these patients were hemizygous, at least for *ENG.*

### Characterization of the breakpoints for the *ENG* deletion mutants

In all these families there were common properties that made them especially interesting for further analyses: all of them contained deletions in *ENG*, not previously published in HHT literature, and all these deletions started on one side of the promoter region of *ENG*. Thus, it was of interest to analyze: i) the mapping of the different deletions; ii) whether there were common breakpoints; and iii) whether there was a common characteristic shared by these regions prone to breaking. Accordingly, breakpoints were studied with a custom designed tiling CGH microarray (Agilent) to detect copy number variations.

**Figure 3:**
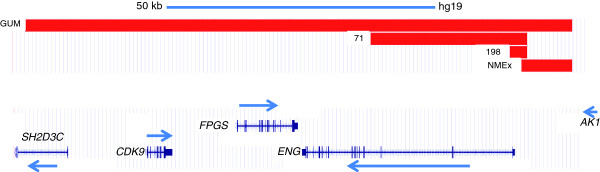
**Schematic representation of the hybridization results of the different HHT1 families containing deletions in chromosome 9q34.11.** In 198, a 3,139-Kb deletion encompassing promoter and exon 1 of *ENG*; in 71: a 28,744-Kb deletion including promoter and exons 1 to 3 of *ENG*. In GUM a 100,730 Kb deletion covering from the proximal promoter of *ENG*, the whole *ENG* gene*,* and *FPGS, CDK9*, *SH2D3C* genes. In NMEx, a 9,313-Kb deletion of *ENG* promoter. Arrows mark the direction of transcription of the genes.

A schematic representation of the final deletion map, for each family, and the genes affected is shown in Figure 3.

A more detailed view of the individual hybridization of each DNA sample to the probes of the array can be seen in Additional file 1: Figure S1. The upper part of the panels in Additional file 1: Figure S1 represents a general view of the hybridization signals within the 9q34.11 region. At the bottom, a zoom of the region containing the copy number alteration is presented in more detail. A shadow box over a crowd of points represents the area where the algorithm has detected a copy number alteration. A minimum of 5 probes, hybridizing in the same manner and placed in the same area, are required to define a significant copy number alteration over a chromosome position.

Table 2 shows a summary of the results obtained from the microarray, showing the exact breakpoints for each deletion found in the four different families. Deletions affecting coding regions of *ENG* include: i) the smallest deletion of 3,139-Kb, affecting the proximal promoter and exon 1 of *ENG* (family 198); ii) an intermediate size deletion encompassing 28,744-Kb, starting at the same point of the proximal promoter as the previous one, and spanning exons 1 to 3 of *ENG* (family 71); and iii) the largest deletion, spanning from 9 kb upstream from the transcription start site of *ENG*, the whole *ENG* gene, and two adjacent *ENG*-downstream genes *FPGS, CDK9* and, at least, partially *SH2D3C* (family GUM). Furthermore, a deletion affecting only the upstream region of *ENG*, which does not include any coding region, comprises 9,313-Kb, from the same position as in the case of the largest deletion of the GUM family in the distal promoter region to position -28 bp before the transcription start for *ENG* (family NMEx).

**Table 2:** Deletion break-points for HHT families

**Sample**	**Cytoband**	**Ins/Del**	**Molecular position**	**Size**	**Regions deleted**	**Affected genes**
JGC	9q34.11	Del	130,617,254-130,626,567	9,313 Kb	Promoter 5' upstream *ENG*	Promoter *ENG*
198/01/11	9q34.11	Del	130,615,100-130,618,239	3,139 Kb	Promoter and Exon 1 *ENG*	*ENG*
71/03/11	9q34.11	Del	130,589,495-130,618,239	28,744 Kb	Promoter and Exon 1–3 *ENG*	*ENG*
MCVD	9q34.11	Del	130,525,837-130,626,567	100,730 Kb	9.3 Kb promoter 5’ upstream, whole *ENG* and surroundings	*SH2D3C, FPGS, CDK9c ENG*

Interestingly enough, these 4 different deletions have some breakpoints in common: two of them start 9.3-Kb upstream of the *ENG* transcription start site, and the other two start approximately 900-bp upstream from the transcription start site. These findings support the existence of “hot-spots” for chromosomal rearrangements in these regions.

**Figure 4:**
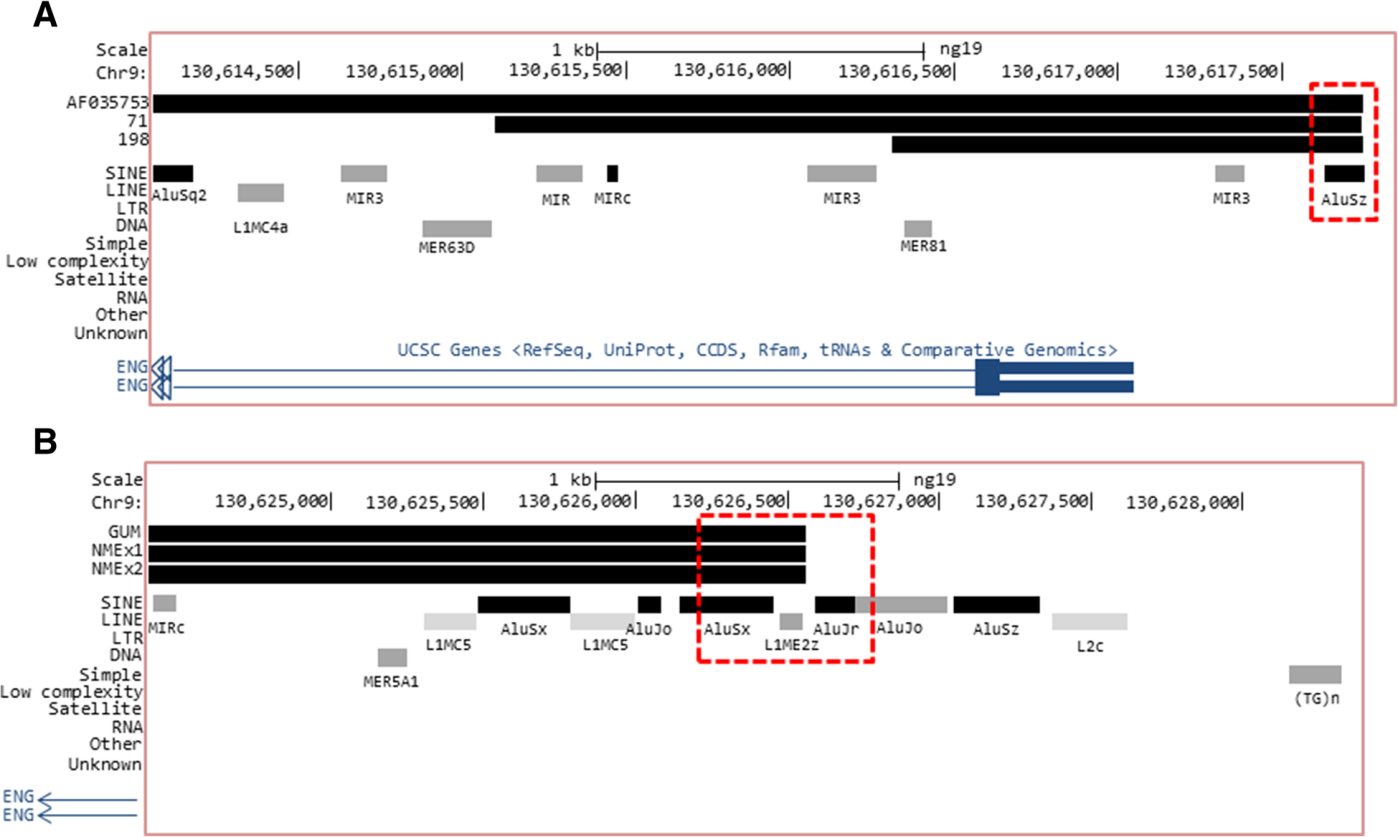
**Map of the common breakpoints for the independent deletions found in the different families. A)** Map for the families 71 and 198, located around 900-bp upstream of the transcription start of *ENG* gene. Repetitive element sequences, including Alu elements, found in this region are represented. **B)** Map of the common breakpoint for the independent deletions found in families NMEx and GUM, placed 9.3-Kb upstream from the transcription start of *ENG* gene. Repetitive elements and Alu sequences are shown. Note that transcription of *ENG* occurs from right to left.

To characterize the nature of these regions prone to “breaking”, we studied the sequences surrounding the common breakpoints for the deletions, on the assumption that regions containing repeated elements are more likely to break. In order to get more insight into the sequence structure of the breakpoints we analyzed these regions in more detail with the Repeat Masker tool for repeat sequences of the UCSC Genome Browser. Figure 4A shows different repeat elements around the breakpoint common to families 198 and 71. Among them, the Alu element AluSz, that contains the common breakpoint, is located 900-bp upstream of the transcription start, and was first described for the *ENG* promoter by Rius et al. [24].

The other breakpoint, found in the remaining two families (GUM and NMEx), is located further upstream from the *ENG* promoter. In this case, there is an accumulation of repeat elements surrounding the breakpoint, including mostly Alu sequences and also a LINE1 element (L1ME2z), as shown in Figure 4B. These results suggest that breakpoints upstream of *ENG* are positioned where mobile or repeated elements are located.

## Discussion

Most of the mutations involved in the pathogenesis of HHT are single base-pair substitutions or small duplications/deletions. In fact, a review of molecular diagnosis of HHT identified for *ENG*, 17% nonsense, 30% missense, 25% splice, and 28% frameshift mutations, whereas for *ACVRL1* 17% were nonsense 60% missense, 7% splice, and 15% frameshift mutations [25].

When no mutations are found in either *ENG* or *ACVRL1* by PCR and sequencing of coding exons and intron boundaries, then the multiplex quantitative PCR (qPCR)-based assay method MLPA is the standard routine technique applied to samples. MLPA allows the identification of dosage for proximal promoter and exons in the HHT-related genes, including deletions or duplications of whole exons. However, this method does not yield a systematic mapping of the breakpoints corresponding to these chromosomal aberrations. So far, mutations affecting the copy number variation of *ENG* or *ACVRL1* causing HHT, as evidenced by MLPA, represent around 7% of all the pathogenic mutations detected in these genes [25,26].

More recently, CNV custom CGH arrays have emerged as an alternative technique to MLPA to detect large deletions or insertions, with the advantage of allowing the mapping and detailed analysis of breakpoints. Thus, this technique can reveal the presence of “hot spots” or points prone to breaking, which may lead to common mutation events in independent families, and coincident with repetitive sequences. In the present work we have identified and mapped four large deletions affecting *ENG* that have not been previously reported in publications and which were first detected by MLPA. To the best of our knowledge, this is the first time that the breakpoints of large deletions in *ENG* have been mapped in independent HHT families.

In all cases the deletions started in the region of the *ENG* promoter and were the first reported deletions affecting the promoter and implicated in HHT. Interestingly, the affected members of the family NMEx were heterozygous for a 9-Kb deletion of the promoter expanding up to 28 bp upstream of the transcription initiation start site. In this case, the loss of the promoter region leading to a hemizygous allele is the likely cause of HHT. Indeed, the proximal promoter region of *ENG* plays a critical role in the basal transcription, mainly through binding of the transcription factor Sp1 to consensus GC-rich motifs [24,27]. Additionally, the promoter activity of *ENG* can be regulated by several physiological stimuli. In this regard, Sp1 can directly bind, in protein-DNA complexes, to the transcription factors KLF6, Smad3/Smad4 or HIF-1α, activated by vascular injury [28], treatment with TGF-β1 [27] or hypoxia [29], respectively. In turn, the multicomplex formed by all these transcription factors leads to synergistic transcriptional cooperation with the promoter activity of *ENG*. Therefore, basal and stimuli-dependent transcription of *ENG* would be abrogated in those HHT patients harboring a promoter deletion, contributing to the haploinsufficiency of *ENG*.

HHT patients from family GUM are hemizygous not only for *ENG*, but also for the genes *FPGS*, *CDK9* immediately downstream, and at least part of *SH2D3C*. However, the additional heterozygous deletion of these genes does not seem to affect the severity or type of clinical symptoms. This would explain the lack of a reported pathology associated with heterozygous mutations of these genes in relevant scientific publications.

The detailed mapping of the deletions has revealed interesting hot spots in *ENG* introns and their upstream promoter region where common breakpoints have been found. Thus, we have identified two different breakpoints placed in non-coding regions corresponding to allocation sites for Alu mobile elements, and both derived from independent recombination events in different families. One of the breakpoints is within a cluster of Alu sequences, 9-Kb upstream of the *ENG* transcription start site (Figure 4B), and the other is on an Alu element, placed 900 bp upstream of the *ENG* transcription start site (Figure 4A). In agreement with this finding, Wooderchak et al. [30] described the breakpoints for two deletions affecting *ENG* in a single HHT family, one of them encompassing exon 3 and the other involving exons 4 to 7 [30]. Interestingly, both deletions share a common breakpoint location in intron 3. Furthermore, a large 117-bp repetitive DNA sequence was identified near the breakpoints in introns 2, 3, and 7 of *ENG*. These repetitive sequences had a sequence identity of approximately 85%, had similar orientation, and were each found to contain Alu elements. These results suggest that this type of mobile element is a common target of recombination in HHT genes. Supporting this view, Alu sequences have been involved in the generation of genomic deletions in different human genetic disorders [31,32]. Further studies have yet to be carried out to better understand the mechanisms of recombination in HHT. In this regard, it would be interesting to screen the published deletions and duplications found in *ENG* and *ACVRL1*, taking advantage of the CNV array technique to obtain a map of deletions or duplications from as little as 15-bp to many kilobases.

## Conclusions

The systematic hybridization of DNA from HHT families, with deletions or duplications, to custom designed microarrays, could allow the mapping of breakpoints, coincident with repetitive Alu sequences that might act as “hot spots” in the development of chromosomal anomalies. New data, generated this way, would then enrich the HHT mutation databank by including the specific sequences of the chromosomal rearrangements, in addition to the current mutations.

## Competing interests

The authors declare that they have no competing interest.

## Authors’ contributions

AF: most sequencing and MLPA. JLF-L: direction of the sequencing and MLPA. RZ: clinician of the hospital center for HHT. LR-P: DNA extraction, PCRs, probe preparation. VA: DNA extraction, and PCR. MLO-F: DNA extraction and PCR. CB: part of the funding support, edition of Ms. LAA: hybridization of CNV arrays. ^*^LMB: direction and coordination of experiments, design and experimental approach strategies. Project fund provision. Writing the manuscript. All authors read and approved the final manuscript.

## Pre-publication history

The pre-publication history for this paper can be accessed here:

http://www.biomedcentral.com/1471-2350/14/121/prepub

## Supplementary Material

Additional file 1: Figure S1 Hybridization results of the different DNAs to the CGH/CNV array. **A)** Hybridization results from sample 198/01/11: 3,139-Kb deletion chromosome 9q34.11: promoter and exon 1 of *ENG*. **B)** Hybridization results from sample 71/03/11, a 28,744-Kb deletion chromosome 9q34.11, including promoter and exons 1 to 3 of *ENG*. **C)** Hybridization results from sample GUM: 100,730-Kb deletion chromosome 9q34.11: promoter *ENG, FPGS, CDK9* and *SH2D3C* genes. **D)** Hybridization results from sample NMEx: 9,313-Kb deletion chromosome 9q34.11: promoter of *ENG*. In all cases, the general view of chromosome 9 with the probe hybridization is presented above, and below the region containing the deletion is magnified to show details. Click here for file
