# Supplementary figures and images for: Copy number variations in endoglin locus: mapping of large deletions in Spanish families with hereditary hemorrhagic telangiectasia type 1
Source: BMC Med Genet. 2013 Nov 25;14:121. doi: 10.1186/1471-2350-14-121 (PMC4222255; doi:10.1186/1471-2350-14-121)

## Slide 1
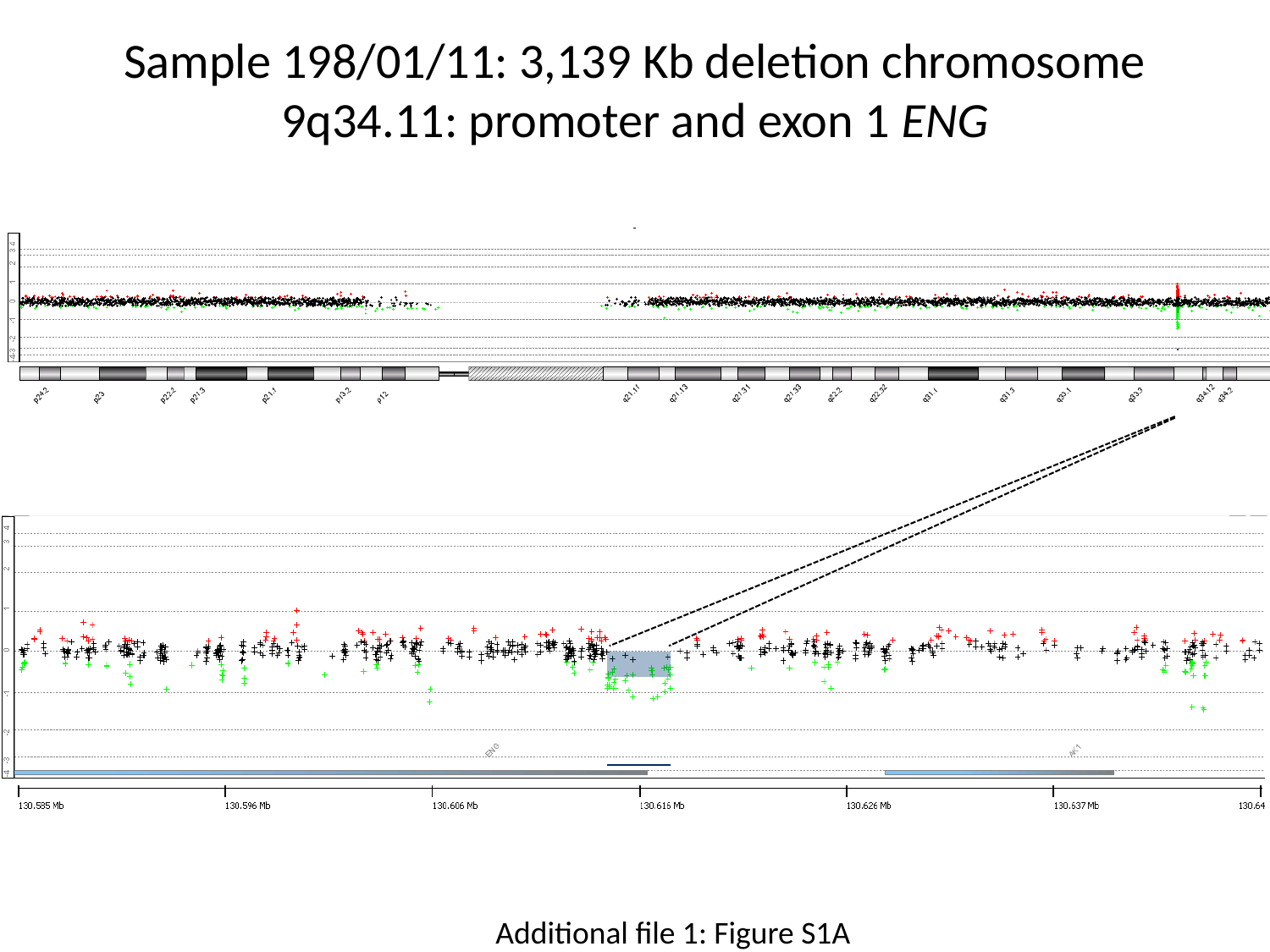

## Slide 2
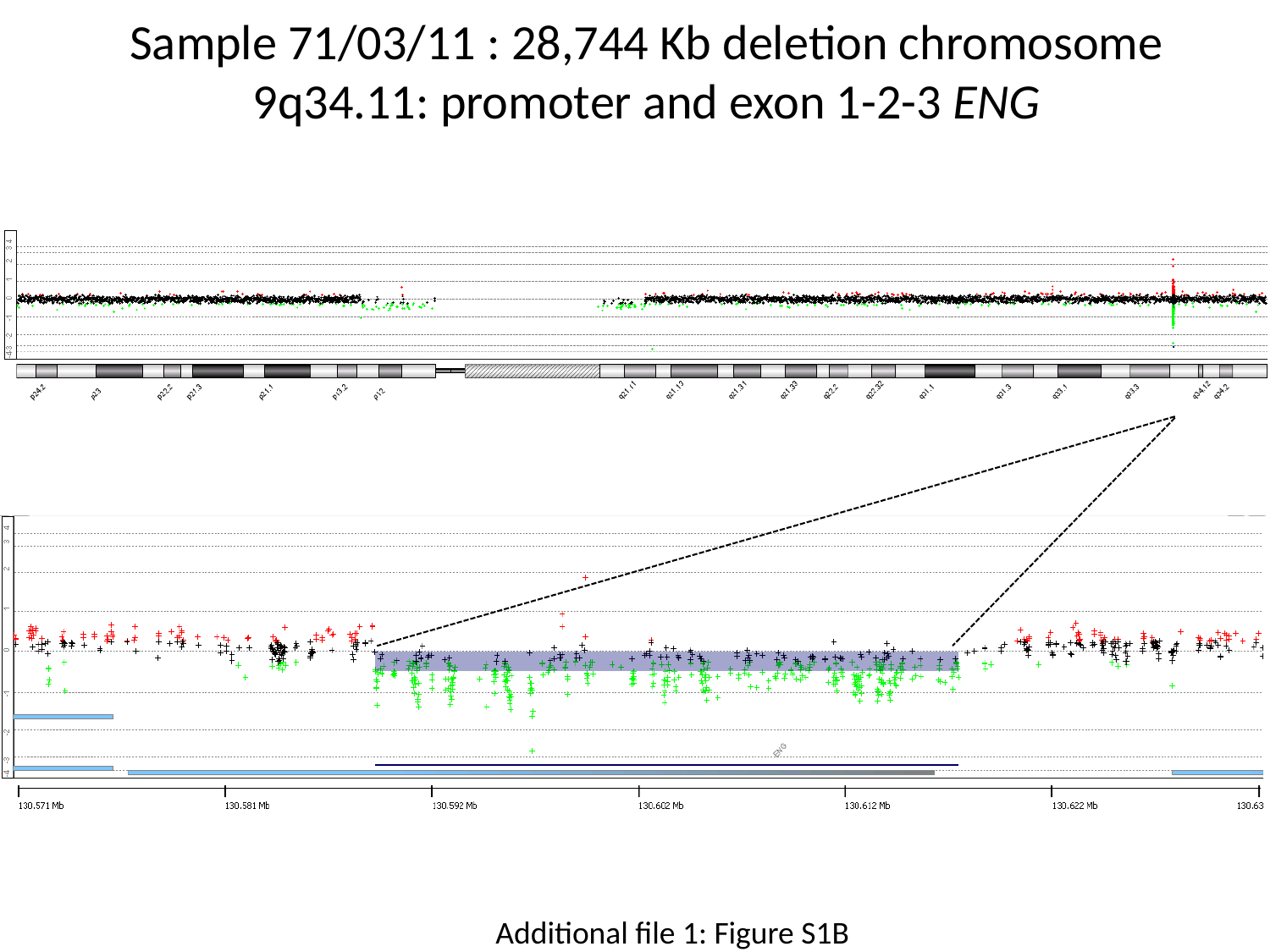

## Slide 3
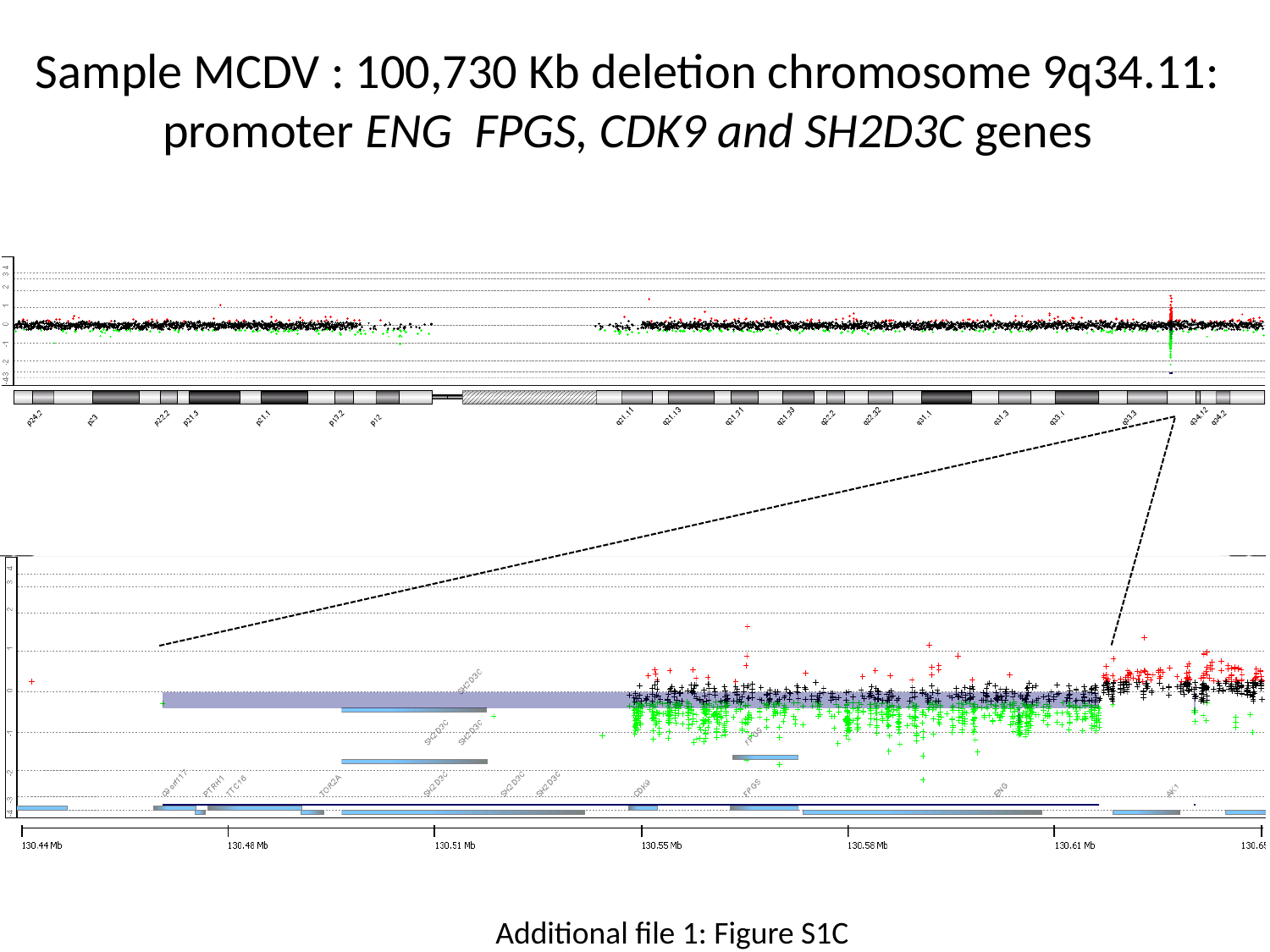

## Slide 4
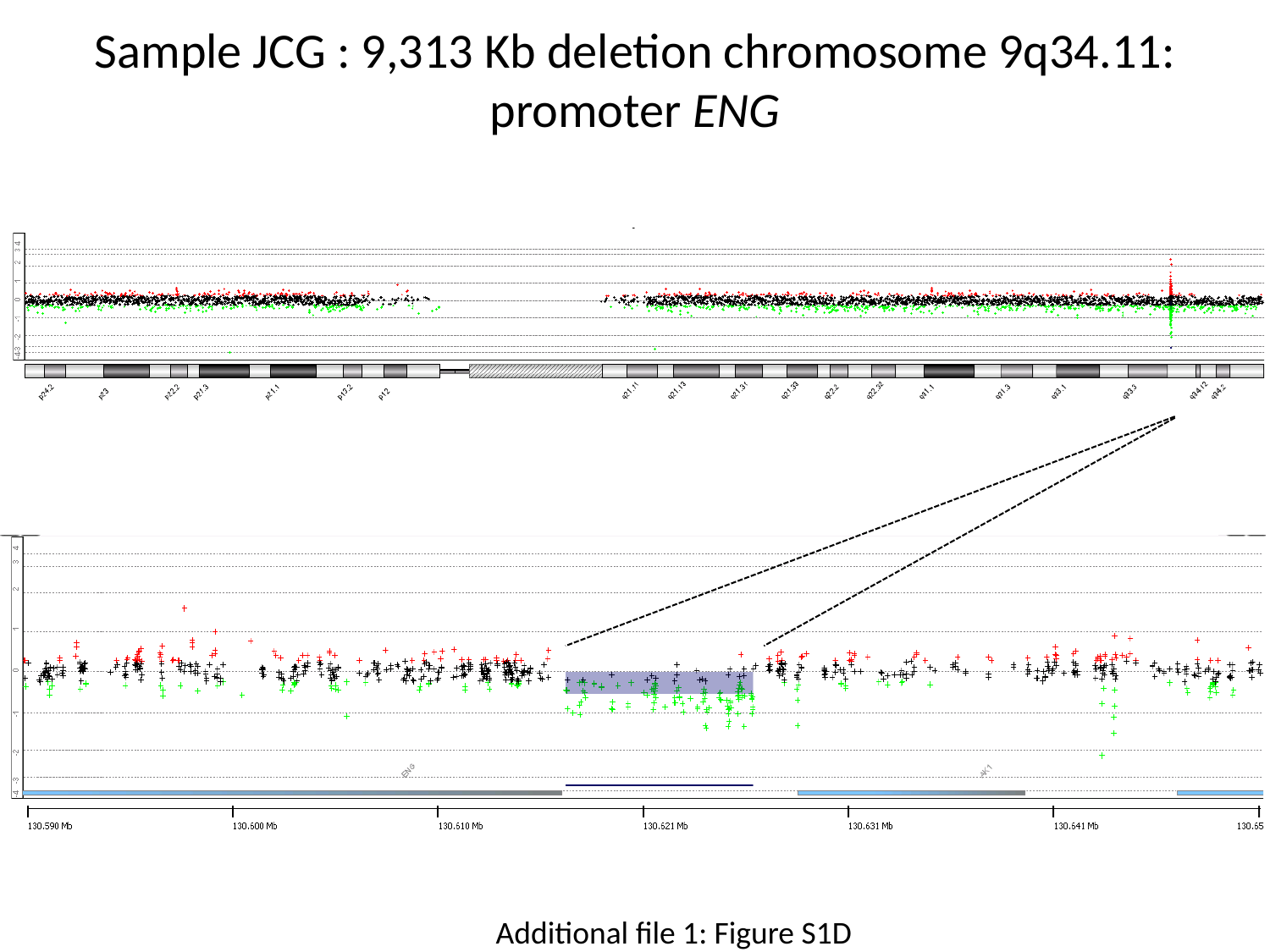

Supplement: Additional file 1: Figure S1 — Hybridization results of the different DNAs to the CGH/CNV array. A) Hybridization results from sample 198/01/11: 3,139-Kb deletion chromosome 9q34.11: promoter and exon 1 of ENG. B) Hybridization results from sample 71/03/11, a 28,744-Kb deletion chromosome 9q34.11, including promoter and exons 1 to 3 of ENG. C) Hybridization results from sample GUM: 100,730-Kb deletion chromosome 9q34.11: promoter ENG, FPGS, CDK9 and SH2D3C genes. D) Hybridization results from sample NMEx: 9,313-Kb deletion chromosome 9q34.11: promoter of ENG. In all cases, the general view of chromosome 9 with the probe hybridization is presented above, and below the region containing the deletion is magnified to show details. [file 1471-2350-14-121-S1.pptx]
